# Timing of restricted sleep: mood and neurobehavioral outcomes in healthy sleepers

**DOI:** 10.1093/sleepadvances/zpad018

**Published:** 2023-03-15

**Authors:** Rammy Dang, Xiaoming Feng, Monika Haack, Janet M Mullington

**Affiliations:** Department of Neurology, Beth Israel Deaconess Medical Center, Harvard Medical School, Boston, MA, USA; Department of Neurology, Beth Israel Deaconess Medical Center, Harvard Medical School, Boston, MA, USA; Department of Neurology, Beth Israel Deaconess Medical Center, Harvard Medical School, Boston, MA, USA; Department of Neurology, Beth Israel Deaconess Medical Center, Harvard Medical School, Boston, MA, USA

**Keywords:** sleep restriction, neurobehavioral outputs, psychomotor vigilance task, mood, visual analog scale

## Abstract

**Study Objective:**

To evaluate how nocturnal timing of sleep restriction affects vigilant attention and mood in healthy controls with normal sleep–wake patterns.

**Methods:**

A convenience sample from two controlled sleep restriction protocols were used to investigate the difference between 4 hours of sleep early in the night, versus 4 hours late in the night. Volunteers stayed in a hospital setting and were randomized to one of the three conditions: a control (8 hours of sleep each night), an early short sleep (ESS, 2300–0300 hours), and a late short sleep (LSS, 0300–0700 hours). Participants were evaluated with psychomotor vigilance task (PVT) and mood ratings via visual analog scales.

**Results:**

Short sleep conditions led to greater performance decrements than control on PVT. LSS performance impairments were greater than control (lapses, *p* = 0.011; median RT, *p* = 0.029; fastest 10%, *p* = 0.038; reciprocal RT, *p* = 0.014; and reciprocal 10%, *p* = 0.005), but had higher positive mood ratings (*p* = 0.005). LSS also had higher positive mood ratings compared with ESS (*p* < 0.001).

**Conclusions:**

The data underscore the negative mood impact of waking at an adverse circadian phase, for healthy controls. In addition, the paradoxical relationship between mood and performance seen in LSS raises concerns that staying up late and waking at the usual rise time may be rewarding in terms of mood, but nonetheless have performance consequences that may not be fully recognized.

**Statement of Significance**

People generally think they are the best judge when it comes to how tired they feel and how much attention they are able to maintain. In this work, we used a convenience sample of data from two sleep restriction protocols (early and late restriction) in healthy volunteers, to test for a difference in how people feel subjectively and how they perform objectively. The finding that LSS leads to improved mood but deteriorated performance echoes findings of the Dinges group which found that volunteers in controlled studies tend to recalibrate their self-assessment of impairment due to sleep loss, and thus may be effectively blind to their impairment (Van Dongen HP, et al. The cumulative cost of additional wakefulness: dose–response effects on neurobehavioral functions and sleep physiology from chronic sleep restriction and total sleep deprivation [published correction appears in *Sleep*. 2004 Jun 15;27(4):600]. *Sleep.* 2003;**26**(2):117–126. doi:10.1093/sleep/26.2.117). This work has implications for safety in emergency and sustained operations where sleep opportunity is limited.

## Introduction

Cognitive performance and emotional well-being are integral components of everyday functioning and high levels of concentration are required to do work-related tasks like operating vehicles and machinery. Sufficient sleep is an important part of maintaining cognitive performance [1–3] and emotional wellness [4, 5]. The amount of sleep required to maintain mental acuity depends on individual [6] as well as environmental factors [7]. However, the consensus is that less than 7 hours of sleep per night can lead to impaired performance and potentially long-term health issues [8]. Reports documenting the negative impact of reduced sleep duration on cognitive functioning cover a wide range of disciplines and include professions such as airplane pilots, athletes, surgeons, and military personnel [9–12].

While sleep restriction is a common occurrence in daily life, there is very little data comparing the effects of sleep loss at different times of night. It is common for adults to delay bedtime due to entertainment, social commitments, and working to meet deadlines for educational or work purposes. It is also not uncommon for adults to need to get up early in order to get to work on time due to long commutes, or child or other caregiving responsibilities that may require rise times to be advanced. We have conducted highly controlled experimental protocols where we have investigated sleep restriction to approximately 50% of habitual sleep time, in healthy adults who normally sleep the recommended 7–8 hours or more [8]. We here compare the effects of sleep restricted to half the normal duration during the early part of the night compared with sleep restricted to the last part of the habitual sleep period.

## Methods

### Participants and study design

The studies examined in this convenience sample were conducted at the Clinical Research Center at Beth Israel Deaconess Medical Center with approval from the Institutional Review Board for the protection of human participants. Participants were recruited through newspaper advertisements and web listings in the Greater Boston metro area. With informed consent, participants were screened comprehensively and excluded if they had any current or past history of psychiatric, neurological, immune, cardiovascular, or sleep disorders. Other exclusion criteria included history of drug use, including cigarette smoking, within the last 6 months. Participants also had toxicology screens and were only enrolled if they did not have shift work or recent time zone changes resulting from travel within 3 months of study enrollment. Their sleeping habits were evaluated by actigraphy and a 2-week sleep diary, and were only enrolled if they had an average sleep duration between 7 and 9 hours in this period, with a habitual bed period falling between 09:00 pm and 11:00 am. Each participant received monetary compensation for participation in the study.

**Figure 1.**
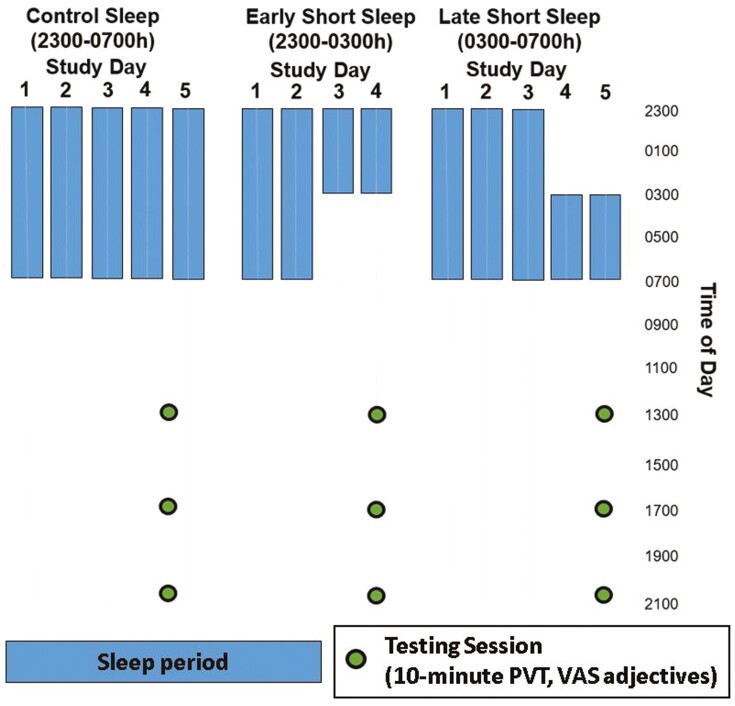
The Control Sleep protocol provided an 8-hour opportunity for sleep each night between 2300 and 0700 hours. Restricted protocols provided a 4-hour sleep opportunity; LSS (0300–0700 hours) and ESS (2300–0300 hours). Participants in each condition had testing sessions after 2 nights of short sleep, that consisted of a 10-minute PVT followed by an adjective assessment battery presented using Visual Analog Scales at 1300, 1700, and 2100 hours. Testing sessions in the pooled control were scheduled at the same clock times but on study day 4 for control sleepers from the ESS study and day 5 for control sleepers from the LSS study (represented by filled dots between days 4 and 5 on the diagram).

Data included from protocols are depicted in Figure 1 (more comprehensive details of protocols are provided in Supplementary Table S1). In the early short sleep (ESS) protocol, participants were randomized to receive 4-hour sleep opportunity from 2300 to 0300 hours or to a control sleep condition (8-hour sleep opportunity from 2300 to 0700 hours). In the late short sleep (LSS) protocol, participants were randomized 4-hour sleep opportunity from 0300 to 0700 hours) or a control sleep condition (8-hour sleep opportunity from 2300 to 0700 hours). In both short sleep conditions, the light levels of participants’ rooms were limited to dim lights (<20 lux) during nighttime waking hours (ESS 0300–0700 hours or LSS 2300–0300 hours) and participants were in semi-recumbent positions during these nighttime waking hours. Before randomization, all participants in both sleep restriction protocols had a baseline period of adjustment in which they slept 8 hours from 2300 to 0700 hours in the hospital bed setting. During their stay in the clinical research center, participants in both protocols followed a regiment of sample collection and testing which included: blood draws, urine collection, psychomotor vigilance task (PVT), and self-reported mood items via visual analog scale (VAS). PVT sessions and VAS mood rating sessions were only included in data analysis at scheduled times which appeared in both the ESS and the LSS protocols because the two sleep restriction protocols differ in testing schedules; ESS have testing sessions every 2 hours, LSS have testing sessions of every 4 hours (Supplementary Table S1). Testing sessions scheduled at times in which participants in one protocol had a difference in sample collection equipment (such as the presence of in-line catheters) were also omitted from analysis to eliminate any influence this difference might have on the results.

### Psychomotor vigilance task

Measures of vigilance and attention were assessed with the PVT-192 device [13]. An LED display showed visual stimuli in the form of increasing time in milliseconds with two push buttons for participants to register their reaction time. Each PVT testing session lasted 10 minutes in duration and was administered in 4-hour intervals at 1300, 1700, and 2100 hours on the day following 2 nights of 4-hour sleep opportunities, which were day 5 of the LSS protocol or day 4 of the ESS protocol. Metrics generated from the PVT equipment per testing sessions included: number of lapses with reaction times of more than 500 milliseconds (ms) (RT > 500ms), median reaction time in ms (Median RT [ms]), fastest 10% of reaction times in ms (Fastest 10% RT [ms]), reciprocal of reaction time (RRT [1/s]), Slowest 10% of reciprocal reaction times (Slowest 10% RRT [1/s]), and mean number of false starts or responses with latencies under 100 ms (RT < 100ms).

Data were aggregated across different testing sessions during the day following the second night of sleep restriction or corresponding day for control participants, and analyzed using mixed model analysis. Time-of-day testing sessions for clock times 1300, 1700, and 2100 hours were also analyzed across groups. In addition, groups were compared for responses with time from waking held constant. Scheduled testing sessions (at 1300, 1700, and 2100 hours) after 2 nights of sleep restriction in either protocol occurred at 6, 10, and 14 hours from wake for control and LSS sleepers; and at 10, 14, and 18 hours from waking time for ESS sleepers. This permitted group comparisons for test that occurred 10 and 14 hours after waking for all groups.

### Visual Analogue Scale—measuring mood variations

Thirty-two adjective items assessing mood were measured using a computerized VAS [14]. Participants used arrow keys on a laptop to move a marker across a graduated scale to report their ratings from “0” to “100” when presented with an adjective item. A list of adjectives items such as “Happy” “Sleepy” or “Angry” were displayed in random sequence to prompt a self-rating response from participants. The adjectives were presented to each participant following the PVT testing. Each scheduled testing session ran for usually less than 15 minutes, including PVT and VAS.

To simplify the analysis of 32 different mood items, ratings from 403 VAS rating sessions from all participants were analyzed using factor analysis in SPSS using principle component analysis combined with varimax rotation [14]. Setting the Eigenvalue cutoff at 1.0 and using a criteria of loading patterns >0.50, qualifying factors were extracted. Mood items that loaded together in one factor were averaged to generate a combined “rating” of each factor for each VAS rating session. These factor ratings were then aggregated across matching rating sessions following the second night of sleep restriction (or control), and analyzed using mixed model analysis. Mean values from each of the testing sessions 1300, 1700, and 2100 hours were compared to examine the effects of the restriction protocols across the three clock time points, and time since waking analyses were conducted on results 10 and 14 hours after waking, as described above for the approach to PVT analyses.

### Statistical analysis

SPSS statistics 28.0 software was used for statistical analysis. Data from participants in the control sleep group of both protocols were pooled and compared with pooled participants from short sleep conditions. Subsequently, data were analyzed for effects of ESS and for LSS compared with control. Each participant was assigned a unique code, which was treated as a random effect, with sleep condition (ESS, LSS, or control sleep) as the fixed effect. Linear mixed models were used to compare each short sleep condition with control; and finally, exploratory analysis of the short sleep conditions with each other. This analysis strategy was applied to all measures.

## Results

### Demographics, PVT, and VAS

Table 1 shows the participant demographics for the 68 participants in short sleep and control conditions. There were no differences between groups in age, BMI, or habitual sleep time (as calculated by 7 days of sleep diary data). Two nights of sleep restriction led to significant performance deficits for the next day, compared with control.

**Table 1.** Estimated means and standard errors of means from linear mixed model analysis of PVT and VAS measures from participants in either control sleep (8 hours) or short sleep condition (4 hours).

	Sleep condition	
	Pooled control sleep (*N* = 33; 16F, 17M)	Pooled short sleep (*N* = 35; 17F, 18M)
Age (years)	28.8 ± 1.3	26.7 ± 1.3
BMI (kg/m^2^)	22.9 ± 0.5	23.7 ± 0.5
Habitual Sleep (h)	8.0 ± 0.10	7.6 ± 0.11
Number of Lapses (RT > 500ms)	2.43 ± 2.10	9.16 ± 2.17^a^
Median Reaction Time (ms)	254.6 ± 25.0	326.3 ± 24.5^a^
Fastest 10% Reaction Time (ms)	197.9 ± 7.7	219.8 ± 7.3^a^
Reciprocal Reaction Time (1/s)	4.01 ± 0.16	3.4 ± 0.16^a^
Reciprocal Slowest 10% Reaction Time (1/s)	2.7 ± 0.16	2.02 ± 0.16^a^
Number of False Starts (RT < 100ms)	5.6 ± 1.5	2.9 ± 1.5
Sleepy (0–100)	33.19 ± 4.8	35.5 ± 4.9
Fatigue (0–100)	25.5 ± 4.7	35.2 ± 5.0

^a^indicates a significant difference (*p* < 0.05) between the pooled short sleep condition and the pooled control sleep condition Well-being.

### Aggregated daytime performance by sleep schedule

**Figure 2.**
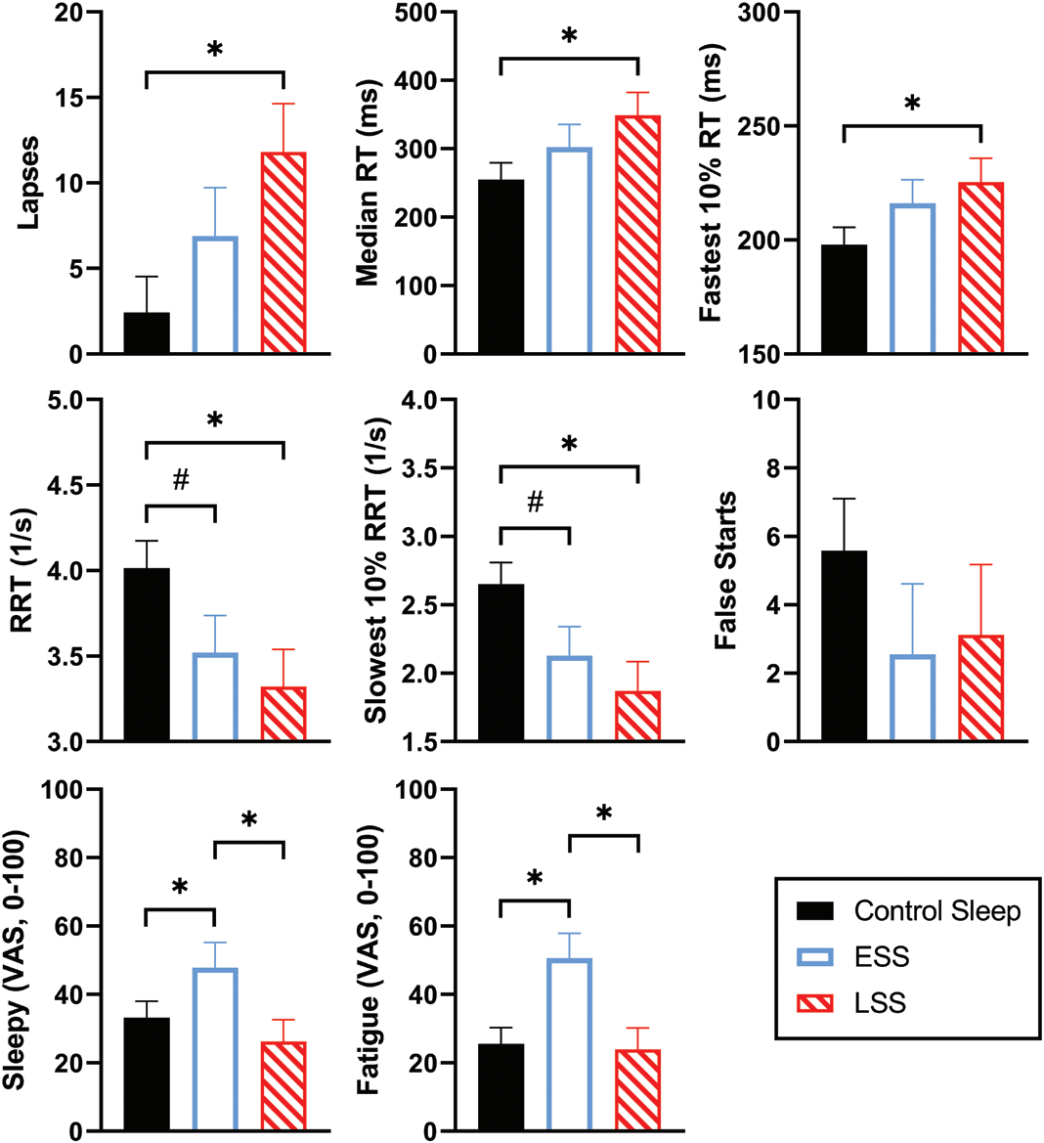
Daytime average of PVT metrics and VAS ratings for Sleepy and Fatigue after two nights of 4-hour sleep restriction in ESS (unfilled bar, 2300–0300 hours) and LSS (hatched bar, 0300–0700 hours), or 8-hour control sleep (filled bar, 2300–0700 hours). Significant differences (*p* < 0.05) are represented with asterisks, and statistical trends (*p* < 0.10) indicated by #. Axis labels are indicated as follows: lapses, indicates number of lapses (RT > 500ms); median reaction time in milliseconds (ms) (Median RT [ms]); fastest 10% of reaction times in milliseconds (Fastest 10% RT [ms]); reciprocal of reaction time (RRT [1/s]); Slowest 10% of reciprocal reaction times (Slowest 10% RRT [1/s]); and mean number of false starts (RT < 100ms).

In analyses of ESS and LSS compared with control (Figure 2), the LSS condition resulted in more impairment on the day’s PVT measures, with significant differences from control for lapse number, median RT, fastest 10% RT, reciprocal RT, and slowest 10% of the reciprocal RT (Figure 2). PVT performance for the ESS condition was not significantly different from the control sleep condition, but there was a statistical trend (*p* < 0.1) towards more impairment in the ESS compared with the control condition on reciprocal RT and reciprocal RT of slowest 10% (Figure 2). Exploratory analyses comparing LSS and ESS conditions found no significant differences in PVT metrics.

ESS participants self-rated significantly higher on the VAS items “Sleepy” and “Fatigued” compared with the control condition, but LSS ratings were not different from control. Moreover, in exploratory analyses, ESS participants rated themselves as more sleepy and fatigued when compared with those in the LSS condition (Figure 2).

### Clock time comparisons

**Figure 3.**
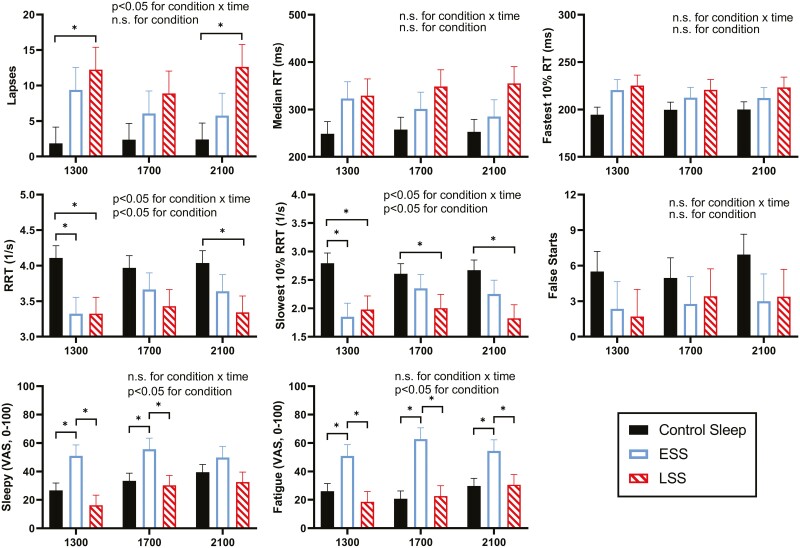
Mean values of PVT metrics and VAS ratings for Sleepy and Fatigue from testing sessions at 1300, 1700, and 2100 hours after two nights of 4-hour sleep restriction in ESS (unfilled bar, 2300–0300 hours) and LSS (hatched bar, 0300–0700 hours), or 8-hour control sleep (filled bar, 2300–0700 hours). Interaction effects are presented, followed by condition tests. Significant differences (p < 0.05) for pairwise tests are represented with asterisks.

(Figure 3): When investigating PVT data broken out by clock times (1300, 1700, and 2100 hours [Figure 3]), ESS only showed significant impairment at one clock time (1300 hours) for slowest 10% RRT and RRT. LSS showed significant PVT impairments at all three test times compared with control for slowest 10% RRT. LSS compared with control also found significant impairment for Lapses and RRT at 1300 and 2100 hours.

ESS rated significantly higher than both control and LSS on “Fatigued” at 1300, 1700, and 2100 hours (Figure 3) and had significantly higher ratings than both control and LSS sleepers on “Sleepy” at 1300 and 1700 hours. LSS sleepers estimated a similar level to control on the VAS items “Sleepy” and “Fatigued,” with no significant differences in any of the three testing sessions.

### Comparisons for time from waking

**Figure 4.**
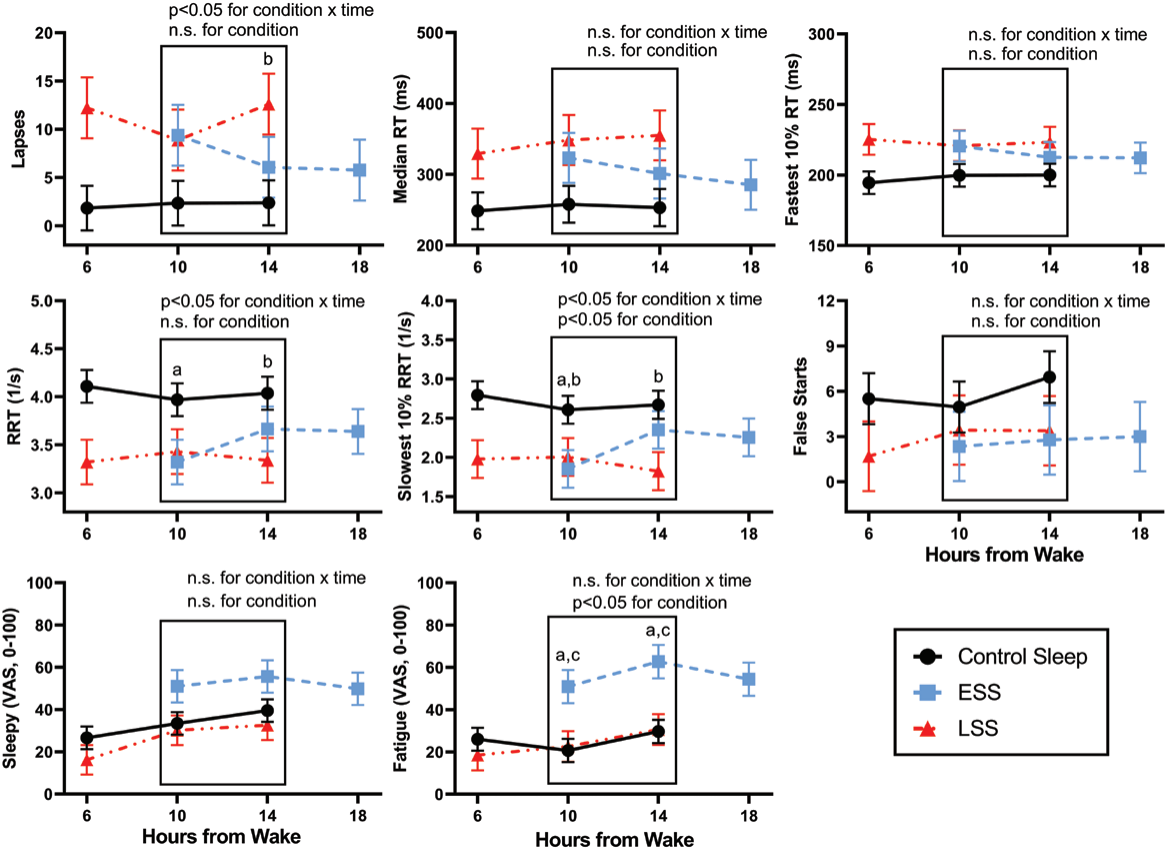
Mean values of PVT metrics and VAS ratings for Sleepy and Fatigue from testing sessions at 1300, 1700, and 2100 hours after 2 nights of 4-hour sleep restriction in ESS (square, 2300–0300 hours) and LSS (triangle, 0300–0700 hours), or 8-hour control sleep (circle, 2300–0700 hours). Values are compared according to the number of hours each group have been awake rather than the actual time of the testing session to account for differences in timing of sleep periods. Only hours 10 and 14 are included in the statistical analysis because these are the only hours at which all three conditions have matching testing sessions. (A) indicates significant (p < 0.05) pairwise differences between ESS and Control. (B) indicates significant (*p* < 0.05) pairwise differences between LSS and Control. (C) indicates significant (*p* < 0.05) pairwise differences between ESS and LSS.

(Figure 4): Aligning PVT and VAS ratings for “Sleepy” and “Fatigued” by number of hours from wake, at 10 and 14 hours after waking (Figure 4) for each group showed consistent results to that of the clock time analyses. ESS sleepers were significantly impaired compared with control on slowest 10% RRT, and RRT at 10 hours after waking. However, LSS sleepers had worse PVT performance than control at both 10 and 14 hours after waking, with greater impairments as reflected by slowest 10% RRT, RRT at 14 hours after waking, and lapses at 14 hours after waking. No significant differences in PVT performance between ESS and LSS were observed at any times from wakening tested.

ESS sleepers rated significantly higher on “Fatigued” at hours 10 and 14, than both control and LSS sleepers (Figure 4). LSS and control sleepers rated at very similar levels for both “Sleepy” and “Fatigued.”

### VAS Adjective Factors–differences in well-being and somatic self-assessments in response to different protocols of sleep restriction

A total of 32 adjective items assessing well-being, mood, and somatic self-ratings by VAS were reduced to five categories based on the results of factor analysis (see Table 2). Quantification of these rated items for each of the five factors were aggregated to generate a composite rating score, which was then analyzed using linear mixed models.

**Table 2.** Results of factor analysis of VAS adjectives. See Statistical Analysis for methodology of factor analysis. Five factors were extracted via principle component analysis. These factors accounted for 63% of the variance in the list of 32 total well-being items shared across the two sleep restriction protocols.

Factor 1: positive mood		Factor 2: pain & discomfort		Factor 3: relaxed & alert		Factor 4: anger & headache		Factor 5: drowsy	
Friendly	0.90	Backache	0.81	Relaxed	0.78	Angry	0.68	Drowsy	0.64
Optimistic	0.86	Hopeless	0.80	Carefree	0.74	Headache	0.58		
Sociable	0.85	Body Pain	0.74	Alert	0.72				
Happy	0.80	Irritable	0.71						
Energetic	0.80	Annoyed	0.67						
Calm	0.80	Stomachache	0.66						
Motivated	0.75	Muscle pain	0.64						
		Discomfort	0.62						
		Lonely	0.61						
No. of Loadings	7		9		3		2		1
Variance Explained (%)	24.30		17.46		10.31		7.49		4.07

**Figure 5.**
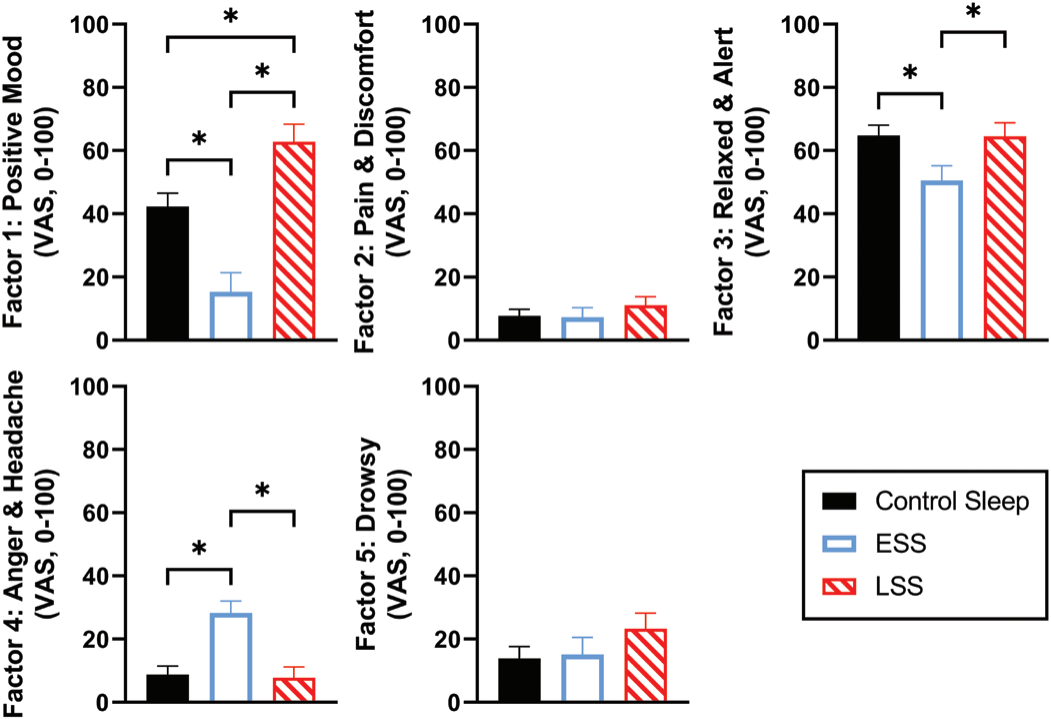
Values of VAS ratings for well-being factors from testing sessions (averaged across test sessions at 1300, 1700, and 2100 hours) after two nights of 4-hour sleep restriction in control sleep (filled bar, 2300–0700 hours), ESS (unfilled bar, 2300–0300 hours) and LSS (hatched bar, 0300–0700 hours). Interaction effects are presented, followed by condition tests. Significant differences (*p* < 0.05) for pairwise tests are represented with asterisks.

Pairwise comparisons were then performed in order to test the effects of restriction protocols on differences in well-being and somatic factors. ESS participants rated significantly lower than both control sleepers and LSS participants on the composite scores for factor 1 and factor 3, which included positive mood items, “Friendly” and “Relaxed” (see Table 2 for a listing of adjectives loading on factors and Figure 5 for the group comparisons of aggregated ratings for each factor). ESS participants rated significantly higher on items in factor 4, with adjectives, “Angry” and “Headache,” when compared to control sleepers and participants in the LSS condition. There were no significant differences between groups in any sleep conditions for factors 2 and 5, which have items relating to physical discomfort and drowsiness. LSS participants rated significantly higher than both ESS participants and control sleepers in the positive mood category (Figure 5).

### Clock time comparisons for factor data

Mean VAS ratings for individual testing sessions at 1300, 1700, and 2100 hours showed similar results to the daytime average of all three sessions. Furthermore, ratings for each well-being factor did not change significantly across the three scheduled testing sessions at 1300, 1700, and 2100 hours (see Supplementary Figure S1).

### Comparisons for time from waking for factor analyzed data

Well-being factors for VAS composite ratings between groups from an hours-from-wake perspective also showed similar results to the daytime averages seen in Figure 5 (see Supplementary Figure S2), with better well-being and mood indices in the LSS when compared with ESS and also when compared with control.

## Discussion

This analysis study using data collected from two very similar, highly controlled experimental protocols of sleep restriction, showed that sleep obtained during the first half of the night (the ESS condition), requiring an earlier rise time by 4 hours, is more burdensome than sleep that is restricted to the second half of the night. Our results in healthy controls demonstrate that waking up at an adverse circadian phase after 2 nights of partial sleep deprivation (PSD) leads to negative outcomes with respect to indices of well-being, including mood, sleepiness, and fatigue. Moreover, while performance impairments were seen in both the ESS and LSS conditions, they were more pronounced in the LSS condition, in spite of the fact that participants had better moods following LSS. These results are consistent with human circadian studies showing that waking up at an adverse phase is associated with performance impairments [15].

Our results are also consistent with the common experience of voluntarily delayed bedtime, where the next day's mood is not very severely impacted. It is common practice, particularly among young adults, to stay up late on the weekend and have to get up early on Monday morning, without adequate recovery. It is not surprising that this pattern results in significant performance impairments, in spite of preserved mood. The mood-improving effect of delayed bedtime (seen in factor 1, “Positive Mood” indices, Figure 5), may explain why this practice is reinforced as a behavior, particularly if it is paired with social activities and there is lack of awareness of performance costs the next day. Curtailing sleep by delaying bedtime to the second half of the night may confer a risk for the morning commute and next day's work performance.

People often base their evaluation of their own performance on how they feel with respect to well-being and mood. The current report adds to the accumulation of evidence that people are not always able to accurately gauge their own performance, and subjective sleepiness and mood indices may asymptote while performance decline continues to accrue [5, 16]. Moreover, these data suggest that the cost of getting up during an adverse phase is subjectively greater than delaying sleep and getting up at the usual time.

Our results can be contrasted with findings from sleep deprivation as a treatment for depression. PSD in the second half of the night (maintaining wakefulness between 0200 and 2200 hours, is of similar effectiveness as total sleep deprivation in alleviating major depressive symptoms; and somewhat better than PSD where sleep is permitted in the latter half of the night [17].

There are several limitations of this work. While the data came from two protocols that were very similar, we could only include test sessions that were aligned in time and free from another coincident testing such as blood draws or blood pressure measures. Nocturnal sleep data via polysomnography was not either not recorded or not available on the night prior to the data points from PVT and adjective items in the ESS protocol. Furthermore, one study had frequent blood draws and the other had beat-to-beat blood pressure, so the sleep could not be examined along with the analyses.

Although we limited the analyses to the most comparable testing sessions between ESS and LSS, differences in testing loads between the two protocols could have potentially influenced the results. ESS participants had a heavier testing schedule with testing occurring more frequently every 2 hours compared to LSS participants who had testing sessions every four hours (Supplementary Table S1). “Testing fatigue” could have contributed to reduced self-reports of well-being and mood, but was probably not the reason for impaired performance, for ESS performance which was not significantly different from control on several measures.

The number of participants in each short sleep condition was small and therefore we were underpowered for mixed model analyses that included all three groups. We were also underpowered for direct comparisons between the ESS and LSS groups. Still, the question about the best time to sleep if amount of time is very limited, such as in emergency operations, is an important one. Understanding the effects of the timing of sleep curtailment, not only on performance indices, but also on mood, an important human factor, is an important area for further research.

While previous sleep restriction research in healthy adult volunteers has shown that repeated ESS leads to increased slow wave activity on the second night of PSD, and also increased power density ratio in REM sleep [18], studies of repeated LSS have reported different patterns. Yang et al. (2017) described reduced REM sleep duration on the second night of LSS, in spite of maintained slow wave sleep duration [19]. Within-participant research designs that include not only an ESS and LSS but also a middle short sleep condition (2-hour delayed and 2-hour advance wake-up) would be informative, particularly if they include clinical polysomnography to enable analysis of electroencephalography and heart rate dynamics in conjunction with performance and mood changes. Understanding the autonomic and cortical state changes and their impact on mood and performance of the next day would provide important information to guide needed sleep curtailment strategies for emergency or sustained operations. Such knowledge would also help in developing evidence-based recommendations for young parents and many others, who may need to cut back on sleep, at times.

## Supplementary Material

zpad018_suppl_Supplementary_Material

## Notes

*This paper is part of the David F. Dinges Festschrift Collection. This collection is sponsored by Pulsar Informatics and the Department of Psychiatry in the Perelman School of Medicine at the University of Pennsylvania.*
