# Supplementary material for: Timing of restricted sleep: mood and neurobehavioral outcomes in healthy sleepers
Source: Sleep Adv. 2023 Mar 15;4(1):zpad018. doi: 10.1093/sleepadvances/zpad018 (PMC10109842; doi:10.1093/sleepadvances/zpad018)
Supplement: zpad018_suppl_Supplementary_Material [file zpad018_suppl_supplementary_material.docx]

|  | ESS Protocol | LSS Protocol |
| --- | --- | --- |
| Duration of study length | 16 days in-hospital stay | 22 days in-hospital stay |
| Screening criteria | - 21-40 years old - 7-9 hours of regular nighttime sleep - no sleep issues confirmed by in-hospital screening PSG - Healthy participants screened with CBC and differentials, thyroid hormones, blood glucose, and urinary toxicology screens | - 25-55 years old - 7-9 hours of regular nighttime sleep - no sleep issues confirmed by in-hospital screening PSG - Healthy participants screened with CBC and differentials, thyroid hormones, blood glucose, and urinary toxicology screens |
| Randomization | After 2 nights of sleep, participants were randomized to:   - control (8-hour sleep) - sustained partial sleep restriction (consecutive 4-hour sleep from study day 3 through study day 13) | After 3 nights of sleep, participants were randomized to:   - control (8-hour sleep) - 4 Cycle Challenge: four cycles with each cycle consisting of three nights of 4-hour sleep (0300-0700) followed by one night of 8-hour sleep (2300-0700) |
| Number of adjective VAS testing sessions  (8-hour sleep days) | On study days 1 and 2: 12 total VAS testing sessions in hourly intervals starting at 0700, except at 0800, 1200, 1400, 1800, 2000, 2200 | On study days 2, 3, 7, 11, 15, 19, 20:  9 total VAS testing sessions in 2-hour intervals starting at 0700 |
| Number of adjective VAS testing sessions  (4-hour sleep restriction days) | On study days 3, **4**, 5, 6, 7, 8, 9, 10, 11, 12:  11 total VAS testing sessions in 2-hour intervals starting at 0300 | On study days 4, **5**, 6, 8, 9, 10, 12, 13, 14, 16, 17, 18:  11 total VAS testing sessions in 2-hour intervals starting at 0700 |
| Number of VAS adjectives in each test session | 108 adjective items | 42 adjective items |
| Number of 10-minute PVT testing sessions  (8-hour sleep days) | On study days 1 and 2:  9 total PVT testing sessions in 2-hour intervals starting at 0700 | On study days **5**, 9, 13, 17:  4 total PVT testing sessions in 4-hour intervals starting at 0900 |
| Number of 10-minute PVT testing sessions  (4-hour sleep restriction days) | On study days 3, **4**, 5, 6, 7, 8, 9, 10, 11, 12, 13, 14, 15:  11 total PVT testing sessions in 2-hour intervals starting at 0300 | On study days **5**, 9, 13, 17:  5 total PVT testing sessions in 4-hour intervals starting at 0900 |
| Injection of placebo or low-dose E-coli endotoxin | Occurs at either:  2300 on study day 11 or 0100 of study day 12 | No injection of placebo or endotoxin |
| Indwelling Catheter | On study day 1:  Participants have an indwelling forearm catheter for 24-hour starting 0900 with hourly blood draws  On study day 11:  Participants have an indwelling forearm catheter starting 0900 with bi-hourly blood draws until the time of injection of placebo/endotoxin. After injection, blood draws will occur at the following frequencies:  1 draw/4 minutes until 30 minutes after injection  1 draw/15 minutes until 0700 of study day 12  1 draw/30 minutes until 1030 of study day 12 | On study days 2, 5, 9, 13, 17, 19:  Participants have an indwelling forearm catheter for 24-hour periods starting 0700 with blood draws every two hours |
| Portapres finger pressure cuffs measuring blood pressure | No finger pressure cuffs | On study days 2, 5, 9, 13, 17, 19:  Finger pressure cuffs for 24-hour |
| Polysomnography (PSG) recording | Not available for the night preceding the analyzed testing sessions. | Not available for the night preceding the analyzed testing sessions. |
| Vascular Reactivity Testing | No Vascular Reactivity Testing | On study days 3, 6, 10, 14, 18, 20:  Occurs at 1600 |
| Rectal thermometer | Participants have a rectal probe measuring body temperature on every study day | No rectal thermometer |
| Urine sample collection | Every study day | Every study day |

Table S1. Comparison of key parts of ESS and LSS protocols. Corresponding study days from either protocols which were compared in PVT and mood analyses are underlined and bolded in the table.

Supplemental Figure S1. Mean values of VAS ratings for mood factors from testing sessions at 1300, 1700, and 2100h after two nights of 4-hour sleep restriction in ESS (unfilled bar, 2300-0300h) and LSS (hatched bar, 0300-0700h), or 8-hour control sleep (filled bar, 2300-0700h). * indicates significant pairwise (p<0.05) differences.

Supplemental Figure S2. Mean values of VAS ratings for mood factors from testing sessions at 1300, 1700, and 2100h after two nights of 4-hour sleep restriction in ESS (square, 2300-0300h) and LSS (triangle, 0300-0700h), or 8-hour control sleep (circle, 2300-0700h). Values are compared according to the number of hours each group have been awake rather than the actual time of the testing session to account for differences in timing of sleep periods. Only hours 10 and 14 are included in the statistical analysis because these are the only hours at which all three conditions have matching testing sessions.

a indicates significant pairwise (p<0.05) differences between ESS and Control

b indicates significant pairwise (p<0.05) differences between LSS and Control

c indicates significant pairwise (p<0.05) differences between ESS and LSS
